# Genetic relatedness and morphology as drivers of interspecific dominance hierarchy in hummingbirds

**DOI:** 10.7717/peerj.13331

**Published:** 2022-04-20

**Authors:** Ubaldo Márquez-Luna, Carlos Lara, Pablo Corcuera, Pedro Luis Valverde

**Affiliations:** 1 Doctorado en Ciencias Biológicas y de la Salud, Universidad Autónoma Metropolitana, Iztapalapa, Ciudad de México, México; 2 Centro de Investigación en Ciencias Biológicas, Universidad Autónoma de Tlaxcala, San Felipe Ixtacuixtla, Tlaxcala, México; 3 Departamento de Biología, Universidad Autónoma Metropolitana, Iztapalapa, Ciudad de México, México

**Keywords:** Trochilidae, Territoriality, Agonistic behavior, Foraging strategies, Dominance hierarchy

## Abstract

A dominance hierarchy is the set of ranks occupied by species within an assemblage. Species with a high position within the dominance hierarchy tend to dominate subordinate species in contests for access to resources. In hummingbirds, greater weight and wing disc loading have been associated with highest ranks within the dominance hierarchy. Nevertheless, the limit to which the difference between the weight of contending species represents a competitive advantage has not yet been determined. Here, we determined the dominance hierarchy of a hummingbird assemblage exploiting the most abundant floral resource (*Palicourea padifolia*, Rubiaceae) in a cloud forest of central Veracruz, Mexico. Specifically, we tested whether species weight and wing disc loading influence the dominance hierarchy. Additionally, we tested whether the flowers visited per foraging bout increases with species weight and dominance. We further tested whether weight, wing disc loading, and the genetic relatedness between contenders influenced the dominance relationships in species-pair interactions. Our results indicate that the hierarchy is positively influenced by weight. Hummingbirds visited similar number of flowers regardless their weight or their dominance. Nevertheless, the probability that the heaviest contender won contests was positively associated with the differences of weight and genetic relatedness between contenders. Contrarily, the probability that the contender with greatest wing disc loading won contests was positively associated with differences of weight and negatively associated with the relatedness between contenders. However, these models only explained between 22% and 34% of the variation, respectively. Our results demonstrate that the weight was the major contributor to high dominance values. However, future studies should include (1) the temporal variability of the weight and (2) experimental predictor variables such the burst power of the hummingbirds to evaluate its effects on the dynamics of dominance hierarchies in hummingbird assemblages. All the hummingbird species present in the studied assemblage have developed wide behavioral mechanisms that compensate their morphological differences, which allow them to coexist, even when they compete for the access to the same resource.

## Introduction

The role of competition for access to food resources by hummingbirds (Wolf, Stiles & Hainsworth, 1976; Graham et al., 2009; Lara et al., 2011) has been suggested as the main factor that influences the species composition and structure on humid and low elevation communities (but environmental filters can act as drivers at higher elevation communities; (Graham et al., 2009). Hummingbirds have to feed on flower nectar from a large number of plants to meet their energy demands generated by their body size, mode of flight, and high metabolic rate (Montgomerie & Gass, 1981; Cotton, 2007). As a consequence, resource segregation is often observed among sympatric hummingbird species. This segregation can occur where flowering periods of different resources are synchronous (López-Segoviano, Bribiesca & Arizmendi, 2017). Segregation can be temporal (Lara, Lumbreras & González, 2009) or can generate differences in the number of flowers visited between dominant and subordinate hummingbirds (Justino, Maryama & Oliveira, 2012).

Dominance is defined as the social position or rank of an individual relative to one or more competitors against which the individual tends to win agonistic contests (Ewald, 1985). A dominance hierarchy is the set of positions or ranks occupied by individuals within a group or species within an assemblage (Chase & Seitz, 2011). The rank of each species can be determined quantitatively by the relative frequency of the contests for resources that it win and lose against contenders (*e.g.*, Rychlik & Zwolak, 2006; López-Segoviano, Bribiesca & Arizmendi, 2017; Francis et al., 2018). The rank in these hierarchies can have repercussions in the health, physiology, weight gain, and reproductive capacity of animals (Chase & Seitz, 2011). Dominant species tend to monopolize the access to high reward resources (Dearborn, 1998). However, the subordinate species have a greater ability to find cryptic or not defended resources (Tiebout III, 1996). For plant species in a community this implies differential rates of flower visitation and pollen transfer that can be compensated by the presence of hummingbirds with different foraging strategies and ranks within the dominance hierarchy.

The outcome of the contests is frequently associated with different morphological traits that provide competitive advantages to the winner species. In hummingbirds, greater weight and higher wing disc loading values have been associated with greater dominance (Kodric-Brown & Brown, 1978; Rodríguez-Flores & Arizmendi, 2016; López-Segoviano, Bribiesca & Arizmendi, 2017; Márquez-Luna et al., 2018; Bribiesca et al., 2019). Wing disc loading is the relationship between wing area relative to body mass. Hummingbirds with smaller wing area relative to body mass will have high wing disc loading (Feinsinger et al., 1979; Feinsinger & Colwell, 1978). This pattern also occurs between sexes in species in which the dominant males have smaller wings than females (Kodric-Brown & Brown, 1978). Wing disc loading values that have been calculated for hummingbirds usually are in a range of 0.02–0.05 g cm^−2^ (Kodric-Brown & Brown, 1978; Feinsinger et al., 1979; López-Segoviano, Bribiesca & Arizmendi, 2017) but the limits of this range depends on the traits of the studied species (weight and wing length). However, high wing disc loading values imply a more energy demand to hover and less energetically efficient flight (Kodric-Brown & Brown, 1978; Feinsinger et al., 1979). On the other hand, species with lower wing disc loading tend to have longer wings relative to weight resulting in an increase of their foraging efficiency (Kodric-Brown & Brown, 1978; Feinsinger et al., 1979). Territorial species tend to have greater wing disc loading and the non-territorial species as the trapliners have lower wing disc loading (Kodric-Brown & Brown, 1978; Feinsinger et al., 1979; Carpenter, Paton & Hixon, 1983). In hummingbird assemblages, the difference between the weight of two contending species can range from several grams (*e.g.*, Blue-throated Mountain-gem, *Lampornis clemenciae* = 8.39 ± 0.12 g and Broad-tailed Hummingbird, *Selasphorus platycercus* 3.74 ± 0.07 g), to less than one gram (*e.g.*, White-eared Hummingbird, *Basilinna leucotis* = 3.95 ± 0.06 g and Broad-tailed Hummingbird = 3.74 ± 0.07 g; Márquez-Luna et al., 2019). In addition, within a species or individual, hummingbird weight can be highly variable; some migratory hummingbird species increase their weight by up to 0.5 g in a single day (Carpenter, Paton & Hixon, 1983). These weight fluctuations could affect the competitive abilities of hummingbirds (Dakin et al., 2018). However, the limit to which the difference in weight between contending species represents a competitive advantage has not yet been determined.

Dominance hierarchies of hummingbirds are dynamic, that is, the rank that the species occupy within the hierarchy is variable. These changes may be associated with the floral resources availability, with seasonal changes in the composition on the hummingbird assemblages, and with preferences on specific floral resources (Rodríguez-Flores & Arizmendi, 2016; Márquez-Luna et al., 2019). This implies that the largest species will not always dominate over the smallest. For example, Martin & Ghalambor (2014) suggests that small sized hummingbird species will be more likely to win contests against larger species or will lose less frequent if both species are distantly related. They suggest that over evolutionary time small species would accumulate morphological or physiological traits that allow them to compensate for the competitive disadvantage of a smaller body size. For example, *Eutoxeres aquila* (weight = 11 g) it is bigger than *Colibri coruscans* (weight = 8 g) but *C. coruscans* have higher muscular capacity (Sargent, Groom & Rico-Guevara, 2021). Additionally, the high levels of steroids regulate the aggressive behavior in the hummingbirds *Sephanoides sephanoides* and *Calypte anna* (González-Gómez et al., 2014). Additionally, between distantly related species there will be greater morphological and physiological variability that can generate a behavioral divergence (Dakin et al., 2018). This divergence can be exploited by small hummingbird species to win contests against larger competitors.

Hummingbird interspecific contests for access to resources generally consist of the following sequence of behavioral events: a hummingbird monopolizes access to a spatially aggregated resource (*e.g.*, a floral patch), this hummingbird (territory owner) perch on few high branches above the floral patch. Subsequently, the owner can use passive defense mechanisms such as vocalizations or visual displays of gorget feathers from the perch or flights around the territory (Baptista & Schuchmann, 1990; Goldberg & Ewald, 1991). These behaviors have a warning role against potential competitors. When other hummingbird (intruder) feeds or approaches to the flowers of the defended patch, the owner begins a chase to expulse the intruder out of the territory. These chases are usually very fast and tend to be won by the hummingbird that owns the territory (Mendiola-Islas et al., 2016). After the chase, the victorious hummingbird returns to its perch and stays vigilant or feeds on the nectar of the defended flowers. Occasionally these chases can escalate to physical contact, and it has been documented that the hummingbirds can use their bills to stab, bite and even pluck feathers from the intruder (Rico-Guevara & Araya-Salas, 2015; Rico-Guevara & Hurme, 2019; Rico-Guevara et al., 2019). Nevertheless, it is difficult to document these physical contacts between hummingbirds under field conditions.

Here, we determined the dominance hierarchy of a hummingbird assemblage exploiting the most abundant floral resource (*Palicourea padifolia*, Rubiaceae) in a cloud forest of central Veracruz, Mexico. Specifically, we tested whether the hummingbird weight and species wing disc loading influence the dominance hierarchy. We hypothesized that the dominant species to be the largest and with the greatest wing disc loading. We also evaluated the flowers visited per foraging as function of species weight and dominance. We expected a positive relationship between weight and the number of flowers visited per foraging bout. Additionally, we expected that dominant hummingbird species would visit more flowers per bout than subordinates. We further explored whether the difference in weight, wing disc loading, and the genetic relatedness between contenders influence the probability that the heaviest hummingbirds and hummingbirds with greatest wing disc loading will win agonistic contests. We expected that the greater the difference between contender traits (weight, wing disc loading and genetic relatedness), the greater the probability that the heaviest hummingbird and hummingbird with greater wing disc loading would win the agonistic contests.

## Materials & Methods

### Study area

Field work was conducted from April to early September, 2017 in the Cloud Forest Sanctuary of the INECOL, A. C. (hereafter CFS), in Xalapa, Veracruz (96°56′16.20″W, 19°30′47.2″N, 1,225 m a.s.l.). We started searching for *Palicourea padifolia* flowers in the study area since April, but flowering was delayed until June. Field work was approved by the administration of the Francisco Javier Clavijero botanical garden. The study area is a 30 ha remnant of cloud forest. The mean annual temperature is 19.3 °C and mean annual precipitation is 1,368.2 mm (Servicio Meteorológico Nacional (SNM), 2019).

### Studied species

*Palicourea padifolia* (Roem. & Schult.) C. M. Taylor and Lorence (Rubiaceae) is a distylous, self-incompatible shrub 2–7 m in height. It is abundant in disturbed areas of cloud forests from southern Mexico to Panama (Taylor, 1989). Individual plants produce 30–40 inflorescences, with 2–4 flowers opening per day during the blooming season, from mid-March to August. The flowering peak is between May and mid-June (Contreras & Ornelas, 1999; Ornelas et al., 2004a). Plants with long-styled flowers (L-morph; corolla length = 14.1 ± 1.35 mm) have shorter corollas than plants with short-styled flowers (S-morph; corolla length = 16.9 ± 1.05 mm), showing a 1:1 morph ratio at the study site (Contreras & Ornelas, 1999; Ornelas et al., 2004a; Hernández-Ramírez, 2018). The number of inflorescences, buds, and open flowers produced by both floral morphs are similar throughout the flowering season (Ornelas et al., 2004a). Flowers from the L-morph produce more nectar but less sugar concentration than S-morph (ranging from 0.59–0.73 µl h^−1^ of nectar volume with a sugar concentration of 15.7–17.5° BRIX). This promotes both floral morphs are equally visited by the hummingbirds in the CFS (Ornelas et al., 2004b). The yellow flowers last a single day, opening just before dawn and wilting at dusk (Ornelas et al., 2004a). Only when the flowers are pollinated by the opposite morph the plants set seeds (Contreras & Ornelas, 1999).

Following the taxonomy proposed by Chesser et al. (2020), eleven species of hummingbirds (Mexican Violetear, *Colibri thalassinus*; Green-breasted Mango, *Anthracothorax prevostii*; Rivoli’s Hummingbird, *Eugenes fulgens*; Amethyst-throated Mountain-gem, *Lampornis amethystinus*; Bumblebee Hummingbird, *Selasphorus heloisa*; Wedge-tailed Sabrewing, *Pampa curvipennis*; Violet Sabrewing, *Campylopterus hemileucurus*; Azure-crowned Hummingbird, *Saucerottia cyanocephala*; Berylline Hummingbird, *S. beryllina*; Buff-bellied Hummingbird, *Amazilia yucatanensis* and White-bellied Emerald, *Chlorestes candida*) and some insects, mainly Hymenopterans and Lepidopterans, visit the flowers of *P. padifolia* (Ornelas et al., 2004a). Wedge-tailed Sabrewing, Azure-crowned Hummingbird and Buff-bellied Hummingbird establish and defend foraging territories around patches of *P. padifolia* (Ornelas et al., 2004a; Jiménez, Negrete-Yankelevich & Macías-Ordóñez, 2012).

### Field procedures

The observations to register defense of foraging patches and agonistic behavior was from 8:00 to 13:00 h, when hummingbirds are more active foraging and nectar production is high in this plant species (Ornelas et al., 2004a). We determined that a floral patch of *P. padifolia* was a defended foraging patch according to the following criteria: (1) the territory owner always returned to the same perch near to the patch, (2) foraged within the patch, and (3) actively defended the patch through chases or vocalizations (Camfield, 2006; Márquez-Luna, Lara & Ortiz-Pulido, 2015; Mendiola-Islas et al., 2016; Márquez-Luna et al., 2019). Because the tiny size of the permanent bands typically used to tag hummingbirds makes it infeasible to recognize them individually in our field observations, we did not tag hummingbirds. Instead, during the behavioral recording each territory owner was identified through its vocal behavior displayed from its perches, since some territorial species exhibit constant vocalizations as part of a passive defense (Goldberg & Ewald, 1991). Therefore, throughout the study we identified the territorial species as the hummingbird species that continuously used active (chases) or passive (vocalizations from a perch) behaviors to defend the foraging patch. All other hummingbird species were categorized as non-territorial.

We were able to monitor 27 floral patches 19 of which met the criteria to be considered as defended foraging patches. Foraging patches consisted of 1–4 aggregated *P. padifolia* plants. These foraging patches were at a distance of at least of 200 m from each other. Each foraging patch was monitored once. A single experienced observer recorded the owner and intruder behaviors during 90 consecutive minutes in each of the identified foraging patches using binoculars (10 × 42). In one day only one or two foraging patches were monitored. In each observation period, we recorded the species identity of the territorial hummingbird, intruders and winner of each interspecific contest. All the contests began within the foraging patch but on many occasions led to chases to expel the intruder outside its limits. Thus, we defined the winner species as the hummingbird that successfully expels the contender outside the boundaries of the foraging patch or at the end of the contest. The hummingbird that won the contest always returned to perch within or near the floral patch or foraged in the defended flowers within the patch. These data were used to determine the agonistic interaction network and the species ranks within the dominance hierarchies.

### Foraging patches of *Paliocourea padifolia* flowers

We counted the number of visited flowers of *P. padifolia* (*i.e.*, the hummingbird introduced its bill inside the flower) per foraging bout by each hummingbird. A foraging bout was defined as the period in which a hummingbird began to visit flowers until the moment when it returned to perch, left the foraging patch or was expelled by another hummingbird. Interspecific competition to resources can promote the segregation of competing individuals. Usually the heaviest species end up monopolizing the best resources (López-Segoviano, Bribiesca & Arizmendi, 2017). However, in the CFS the *P. padifolia* flowers are the main floral resource, so we expected that smaller subordinate species will be sate visiting fewer flower per bout than larger dominant hummingbird species.

We counted the number of open flowers within the foraging patches around the time that each owner was observed. When the foraging patches had many flowers, we took 2–3 photographs from sides of the *P. padifolia* patch that we used to manually count the flowers through multi-point tool of ImageJ software (Schneider, Rasband & Eliceiri, 2012). Due to the spatial aggregation of *P. padifolia* plants we used the number of flowers rather than the patch size as an estimate of the floral patch quality. In addition, high number of flowers within the foraging patch can promote behavioral responses (*e.g.*, more aggressive defense of resources or increased presence of intruders) in the intruders and territorial hummingbird (Dearborn, 1998).

### Estimation of the species ranks within dominance hierarchies

Dominance hierarchies of the CFS hummingbird assemblage were established using two methods: a modified Elo-rating method proposed by Sánchez-Tójar, Schroeder & Farine (2018) and David’s score (David, 1987). Both methods use the agonistic dyadic interactions as input data. However, the Elo-rating is based on the randomization of temporal sequence of the interactions (Sánchez-Tójar, Schroeder & Farine, 2018) and David’s score is calculated through an interaction matrix. In hummingbird assemblages, the outcome of the agonistic interactions between a pair of species can be highly variable. Additionally, there are species that tend to participate more frequently than others in agonistic interactions. These factors are source of uncertainty in the calculation of dominance hierarchies (Sánchez-Tójar, Schroeder & Farine, 2018). Elo-rating randomization provides an estimation of the variability of the species position within the dominance hierarchy through the calculation of 95% confidence intervals (Sánchez-Tójar, Schroeder & Farine, 2018). David’s score considers the proportion of contests won and lost by each pair of species weighted by the total contests between each pair of contending species (David, 1987; Gammell et al., 2003). These two methods can be complementary since they both allow controlling different sources of uncertainty in the estimation of highly variable dominance relationships as occurs in hummingbird assemblages.

The Elo-rating method (Elo, 1978) establishes that the order in which the contests were held affects the inference of the dominance rank of the contestants, since after knowing the result of each contest the contenders’ score is updated (adding points to the winner and subtracting them from the loser). The magnitude of the change in the score of both contenders is established according to the probability that one of the contenders wins the contest, and this probability will change with each match between two specific contenders based on the following equation:

\begin{eqnarray*}pA=1/(1+\exp \nolimits \left( -0.01 \left( EloA-EloB \right) \right) ) \end{eqnarray*}

where *pA* is the probability that the species *A* wins, while *EloA* is the punctuation of the species *A* before the contest and *EloB* indicates the punctuation of the species *B* before the contest. A detailed example of Elo-rating calculation can be found in Sánchez-Tójar, Schroeder & Farine (2018).

Sánchez-Tójar, Schroeder & Farine (2018) modified this method by randomizing the order in which the contests occurred (*n* = 1, 000 randomizations), thereby allowing calculation of an average Elo-rating and 95% confidence intervals. High values of Elo-rating indicate higher ranks within the dominance hierarchy. To calculate the randomized Elo-rating we use the R package aniDom v. 0.1.4 (Farine & Sánchez-Tójar, 2017).

David’s score was calculated with this equation:

\begin{eqnarray*}DS=w+{w}_{2}-l-{l}_{2} \end{eqnarray*}

where *w* is the sum of the contests won by species *i* against species *j* divided by the total of contests between both species, *w*_2_ is the sum of values *w*, *l* represents the sum of the contests won by species *j* against species *i* divided by the total of contests between both species and *l*_2_ represents the sum of values *l*. A detailed example of David’s score calculation can be found in Gammell et al. (2003). High values of David’s score indicate higher ranks within the dominance hierarchy. To calculate the David’s score we used the R package compete v. 3.1.0 (Curley, 2016).

Both methods have been used to determine interspecific dominance hierarchies among birds that competed for access to resources (López-Segoviano, Bribiesca & Arizmendi, 2017; Francis et al., 2018; Márquez-Luna et al., 2019).

### Morphological differences between contenders

To estimate the morphological differences between contenders we used weight and wing length (wing chord) data of 10 species collected in 1999 by CL in the CFS during the flowering season of *P. padifolia* (Table S1; Fig. S1). The hummingbirds were captured during their period of higher activity using mist nets. These morphological data were taken using a vernier caliper and a digital scale. The morphological data comprises measures for both sexes (*n* = 8 males and 7 = females) because we want to include the greatest variability of these traits within each species. Using these data, we calculated the difference in weight and wing disc loading (wdl) between the winner and loser species of each contest. To estimate wing disc loading we used the following equation (Feinsinger et al., 1979):

\begin{eqnarray*}wdl=W/\pi (l+0.404{l}^{0.6})^{2} \end{eqnarray*}

where *W* is the weight in grams and *l* is the wing chord in centimeters.

### Genetic relatedness between contenders

We estimated the genetic relatedness between the species throughout the genetic distance using the Tamura-Nei model (Tamura & Nei, 1993) through MEGA software version 7.0 (Kumar, Stecher & Tamura, 2016). This model estimates the proportion of nucleotide sites at which two sequences compared are different. The values of this estimation can range between close to 0 (when the species are closely related) to 1 (when the species are distantly related). We estimated the genetic distance using sequences available at Genbank (Clark et al., 2016) from the mitochondrial gene NADH subunit 2 (ND2, 1041 bp). We used ND2 gene because it has a high variability over time (mean rate = 0.0081 substitution/per site/million years; Nabholz, Lanfear & Fuchs, 2016). In our study we used complete ND2 sequences for all hummingbird species except for Wedge-tailed Sabrewing (Table S2). Due the sequences available in GenBank for this species were not complete (accession number: KC858426, 393 bp and KC858427, 393 bp), we used a complete sequence of the ND2 gene from the closely related species Long-tailed Sabrewing (*Pampa excellens*). When several genetic sequences were available for each species, we select that with the geographic origin closest to the study region.

### Statistical analysis

Hummingbird interactions were summarized as a bipartite matrix, with each cell filled with the frequency of the pairwise interaction between each hummingbird species. We built an agonistic interaction network to illustrate dominance relations between species and the degree of each species (number of species the target species is linked to, Dormann, Gruber & Fründ, 2008). We also estimated connectance as the proportion of the possible links in the network that are realized using the R package bipartite version 2.15 (Dormann, Gruber & Fründ, 2008). We used multiple linear regression to test whether weight and wing disc loading influence the dominance hierarchy (as measured by Elo-rating and David’s score) of the hummingbird species in the CFS. To avoid the problem of collinearity between weight and wing disc loading (*r* = 0.90, *df* = 97, *P* < 0.0001) we calculated the partial *R*^2^ to determine the relative importance of each variable in the models. Partial *R*^2^ were performed using the R package rsq version 2.2 (Zhang, 2018). In these models the interaction among weight and wing disc loading were tested and dropped from the models if unimportant. To test whether the species weight and dominance (as measured by Elo-rating and David’s score) influence the number of flowers visited per foraging bout, we used linear regressions. We used GLMMs (binomial distribution, logit link function) to tests whether the difference in (1) weight, and (2) wing disc loading, and (3) genetic relatedness between contenders, influenced which species won the contests. In these models we included the floral patch identity and the total number of flowers within the patch as random effects (*i.e.*., 19 foraging patches but 99 recorded contests). In these models we do not included observation time as a variable because we monitor one or two foraging patches daily to warranty that all observations were made within the period of greatest hummingbird activity (8:00 to 13: 00 h; Ornelas et al., 2004a). We tested two binomial response variables (1) when winner is due to weight and (2) when winner is due to wing disc loading. In both response variables the result of each contest were coded as follows: winners were classified as “1”, losers as “0”. Due the highly collinearity between weight and wing disc loading we tested these explanatory variables in different models (Table 1 for models details). In all models the interaction among variables were tested and dropped from the model if unimportant. The GLMMs were constructed in lme4 version 1.1–27.1 (Bates et al., 2015). We select the best model by Akaike’s information criterion and the total variance explained (*R*^2^) by the full model (Zuur et al., 2009). We estimated the *R*^2^ of full models including the random effects and the contribution of each predictor variable using semi-partial *R*^2^ through the package partR2 version 0.9.1 (Stoffel, Nakagawa & Schielzeth, 2021). All statistical analyses were performed in R v.3.5.3 (R Core Team, 2019). The agonistic interaction network and the GLMMs were plotted using R packages igraph version 1.2.1 (Csárdi & Nepusz, 2006) and ggplot2 version 3.3.5 (Wickham, 2016) respectively. Data supporting this study are available in the Open Science Framework: https://osf.io/hsygt/?view_only=18f4896c39fb4182bbea9a4052dfd79e.

**Table 1:** GLMMs details. We tested whether the difference in (1) weight, and (2) wing disc loading (WDL), and (3) genetic relatedness between contenders, influenced which species won the contests. We tested two binomial response variables (1) when winner is due to weight and (2) when winner is due to wing disc loading. Explanatory variables with the symbol Δ indicate difference between contenders. Due to collinearity between weight and wing disc loading we tested these explanatory variables in different models. In all models the interaction among explanatory variables were tested and dropped from the model if unimportant. In all models we included the floral patch identity and the total number of flowers within the patch as random effects.

Model	Response variable	Explanatory variables
1	Winner is due to weight	Δ weight
		genetic relatedness betwen contenders
2	Winner is due to weight	Δ WDL
		genetic relatedness betwen contenders
3	Winner is due to WDL	Δ weight
		genetic relatedness betwen contenders
4	Winner is due to WDL	Δ WDL
		genetic relatedness betwen contenders

## Results

We found 19 foraging patches in 40.5 h of observation during the flowering season of *P. padifolia*. In these floral patches we registered 10 hummingbird species: Mexican Violetear, Green-breasted Mango, Rivoli’s Hummingbird, Amethyst-throated Mountain-gem, Bumblebee Hummingbird, Wedge-tailed Sabrewing, Azure-crowned Hummingbird, Berylline Hummingbird, Buff-bellied Hummingbird and White-bellied Emerald. The territorial species were Rivoli’s Hummingbird (five defended floral patches), Wedge-tailed Sabrewing (five defended floral patches), Azure-crowned Hummingbird (five defended floral patches), Berylline Hummingbird (three defended floral patches) and Buff-bellied Hummingbird (one defended floral patch). The Wedge-tailed Sabrewing most frequently used vocalizations as part of their territorial behavior. The other hummingbird species were non-territorial. We were unable to identify trapliner hummingbirds because our observations were focused on isolates foraging patches. We registered 3904 flowers visited by hummingbirds in 169 foraging bouts. The Azure-crowned Hummingbird made 31% of the visits (*n* = 1193 visited flowers), while Green-breasted Mango visited only 1% (*n* = 45) of the total number of flowers visited by hummingbirds (Table S3). We recorded 149 contests, of which 99 were interspecific and 50 intraspecific.

**Figure 1:**
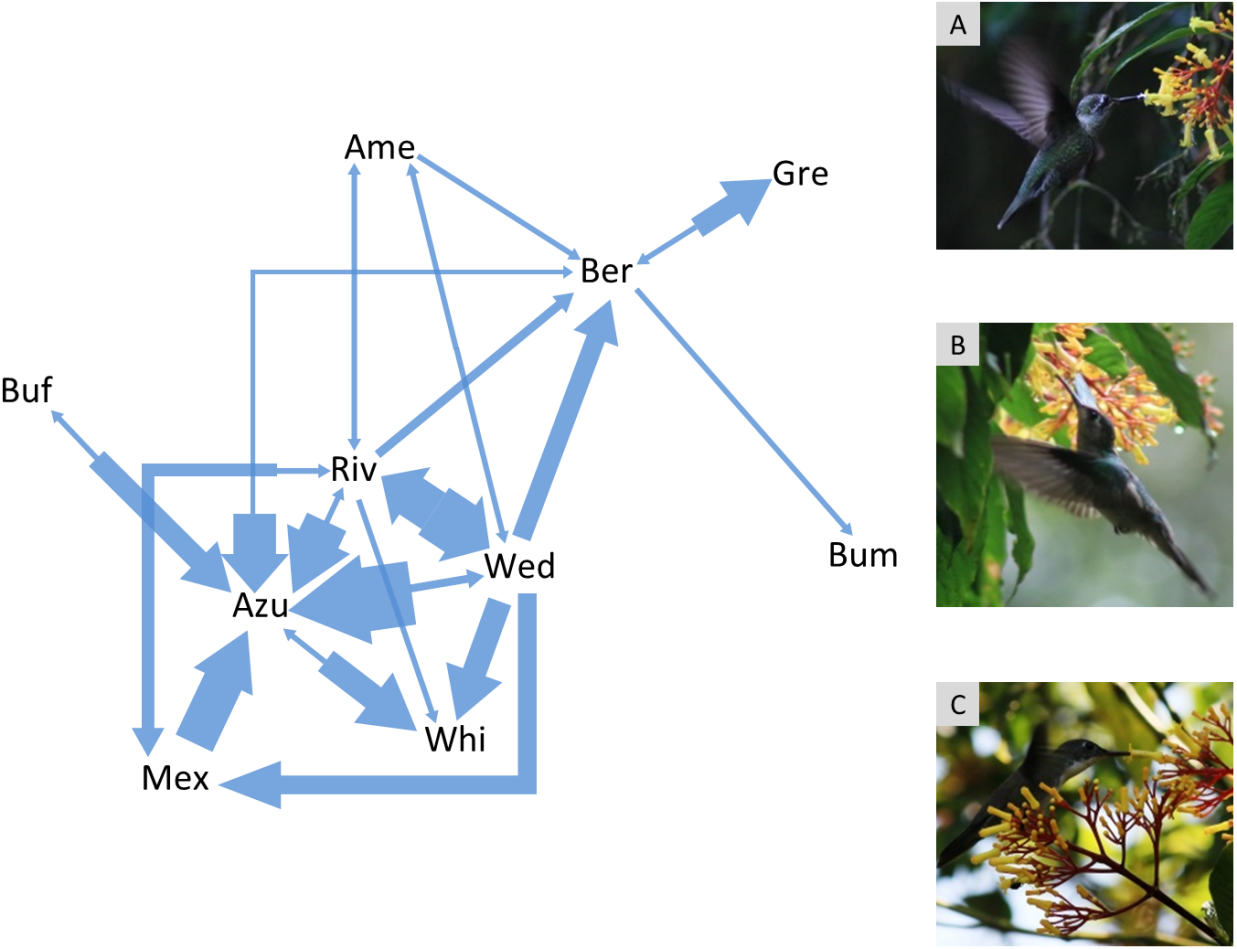
Agonistic interaction network of hummingbird species in the Cloud Forest Sanctuary (CFS). Arrows indicate agonistic interactions between hummingbird species. Each arrow links a pair of contending species; the arrow direction points to the species that lost the contest. The arrow width represents the frequency of contest results between each pair of species. The agonistic interaction network had 31% of the possible links (connectance = 0.31). Gre, Green-breasted Mango (*Anthracothorax prevostii*); Mex, Mexican Violetear (*Colibri thalassinus*); Riv, Rivoli’s Hummingbird (*Eugenes fulgens*); Ame, Amethyst-throated Mountain-gem (*Lampornis amethystinus*); Bum, Bumblebee Hummingbird (*Selasphorus heloisa*); Wed, Wedge-tailed Sabrewing (*Pampa curvipennis*); Azu, Azure-crowned Hummingbird (*Saucerottia cyanocephala*); Ber, Berylline Hummingbird (*Saucerottia beryllina*); Buf, Buff-bellied Hummingbird (*Amazilia yucatanensis*) and Whi, White-bellied Emerald (*Chlorestes candida*). The photographs correspond to the hummingbird species with the highest degree (6 links) within the agonistic interaction network: A = Rivoli’s Hummingbird (immature male), B = Wedge-tailed Sabrewing, C = Azure-crowned Hummingbird. Photo credit: Ubaldo Márquez.

The agonistic interaction network had 31% of the possible pairwise interactions (connectance = 0.31; Fig. 1). Azure-crowned Hummingbird, Wedge-tailed Sabrewing, Rivoli’s Hummingbird and Berylline Hummingbird were the most frequently seen visiting the flowers (Table S3) and they were also the species that participated most frequently in contests and against more species (*i.e.*, highest degree in the agonistic interaction network = 6 pairwise interactions). Bumblebee Hummingbird participated less frequently in contests with just one contest lost against Berylline Hummingbird (*i.e.*, lowest degree in the interaction network = 1 pairwise interaction).

**Figure 2:**
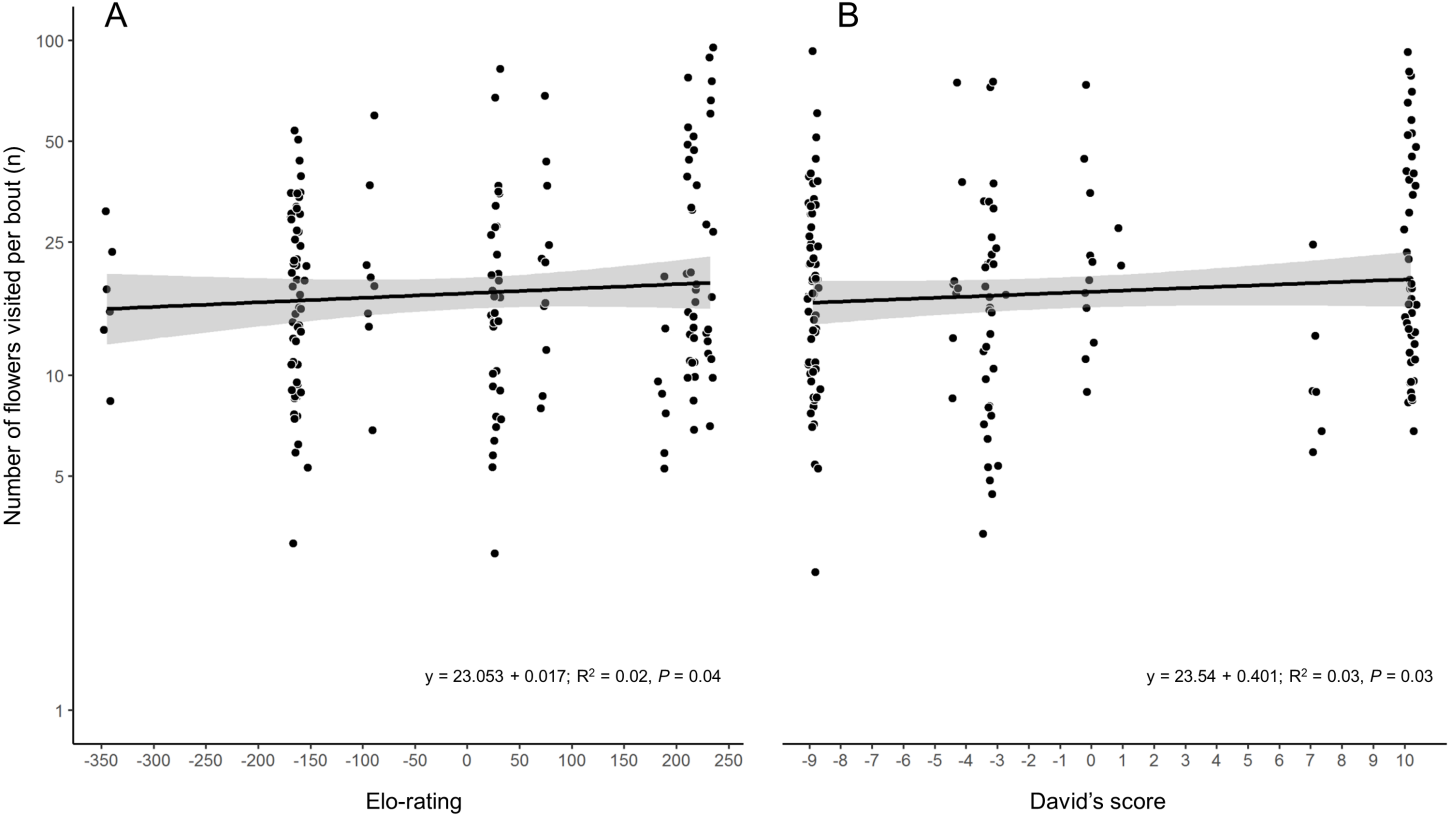
Number of flower visited per foraging bout by each hummingbird species in the CFS. *Palicourea padifolia* flowers visited per foraging bout as a function of the species dominance measured by (A) Elo-rating and (B) David’s Score. The dots were jittered to indicate sample sizes.

While the two hierarchy indexes are strongly correlated (*r* = 0.92, *df* = 8, *P* = 0.0001; Fig. S2), the rank order of some species varied (Table 2). Hummingbird weight ranged from 2.39 g in the Bumblebee Hummingbird to 9.5 g in the Wedge-tailed Sabrewing (Table S1). Wing disc loading varied from 0.0283 g cm^−2^ in the Mexican Violetear to 0.0536 g cm^−2^ in the Wedge-tailed Sabrewing (Table S1). The linear regression suggested that greater weight is the main contributor to dominance measured by Elo-rating and David’s score (Table 3). The linear regressions indicated that hummingbirds visited similar number of flowers regardless their weight (*R*^2^ = 0.01, *F*_1,167_ = 3.17, *P* = 0.07) or their dominance (Fig. 2) measured by Elo-rating (*R*^2^ = 0.02, *F*_1,167_ = 4.10, *P* = 0.04) and David’s score (*R*^2^ = 0.03, *F*_1,167_ = 4.72, *P* = 0.03).

**Table 2:** Hummingbird dominance hierachy. Dominance hierarchies based on Elo rating (Farine & Sánchez-Tójar, 2017) and David’s score (David, 1987).

Hummingbird species		Elo rating/Rank	David score/Rank	Elo 95% CI	Contests	
Common name	Scientific name				W	L
Rivoli’s Hummingbird	*Eugenes fulgens*	232.2/1	10.1/2	1–5	25	11
Wedge-tailed Sabrewing	*Pampa curvipennis*	214.5/2	10.2/1	1–5	32	13
Amethyst-thorated Mountain-gem	*Lampornis amethystinus*	185.5/3	7.2/3	1–4	3	2
Mexican Violetear	*Colibri thalassinus*	73.1/4	−0.1/5	2–6	9	6
Berylline Hummingbird	*Saucerottia beryllina*	27.5/5	−3.3/7	2–8	14	8
Buff-bellied Hummingbird	*Amazilia yucatanensis*	20.6/6	0.8/4	3–7	4	1
Bumblebee Hummingbird	*Selasphorus heloisa*	−91.6/7	−4.3/8	5–9	0	1
Green-breasted Mango	*Anthracothorax prevostii*	−152.9/8	−2.9/6	4–9	1	5
Azure-crowned Hummingbird	*Saucerottia cyanocephala*	−164.1/9	−8.9/10	3–10	10	42
White-bellied Emerald	*Chlorestes candida*	−344.9/10	−8.8/9	8–10	1	10

**Notes.**

W won L lost CI Elo rating 95% confidence intervals

**Table 3:** Results of multiple linear regressions. The Multiple Linear Regression evaluates whether the weight and wing disc loading (WDL) of the winner of each contest influence the dominance hierarchy (as measured by Elo-rating and David’s score). *R*^2^ = variance explained by the full model and partial *r*^2^ = contribution of each predictor variable. Model *R*^2^ is not the sum of partial *r*^2^ values, due to the intercorrelations between the predictor variables.

	Elo-rating				David’s score			
	df	*F*	*P*	R^2^*/partial r*^2^	df	*F*	*P*	R^2^*/partial r*^2^
Full model (Weight + WDL)	2, 96	115.7	<0.0001	0.71	2, 96	261	<0.0001	0.84
Weight	1	177.35	<0.0001	*0.61*	1	429.65	<0.0001	*0.76*
WDL	1	54.03	<0.0001	*0.36*	1	92.32	<0.0001	*0.49*

**Table 4:** Results of generalized linear mixed models. The GLMMs evaluates whether the differences in weight, wing disc loading and relatedness between contenders influence which species won the contest. In model 1 and 2 the response variable was categorized as follows: heaviest hummingbird wins (1) or loses (0) the contest. In model 3 and 4 the response variable was categorized as follows: hummingbird with greatest wing disc loading wins (1) or loses (0) the contest. The symbol Δ indicates difference between contenders for that variable. In all models the interaction among variables were tested and dropped from the model if unimportant. Models are ordered by their R^2^ values.

**Predictor**	Estimate	SE	*z*	P
Model 3 Δ weight + relatedness between contenders (AIC = 111.2, *R*^2^ = 0.34)				
Intercept	0.93	1.01	0.92	0.35
Δ weight	1.005	0.27	3.62	0.0002
Relatedness between contenders	−17.18	6.37	−2.69	0.006
**Random effects**	Variance	SD		
Total number of flowers in the foraging patch	0.63	0.79	–	–
Foraging patch ID	0.61	0.78	–	–
Model 1: Δ weight * relatedness between contenders (AIC = 117, *R*^2^ = 0.22)				
Intercept	−3.81	1.36	−2.79	0.005
Δ weight	2.65	1.17	2.25	0.024
Relatedness between contenders	24.67	8.71	2.83	0.004
Δ weight * relatedness between contenders	13.85	6.81	2.03	0.041
**Random effects**	Variance	SD		
Territory ID	0.33	0.57	–	–
Total number of flowers in the foraging patch	0.008	0.09	–	–
Model 2 Δ wing disc loading + relatedness between contenders (AIC = 121.8, *R*^2^ = 0.11)				
Intercept	−1.69	1.007	−1.68	0.09
Δ wing disc loading	0.68	29.27	0.02	0.98
Relatedness between contenders	15.5	6.01	2.57	0.009
**Random effects**	Variance	SD		
Total number of flowers in the foraging patch	0.4	0.63	–	–
Foraging patch ID	0.53	0.72	–	–
Model 4 Δ wing disc loading + relatedness between contenders (AIC = 129.1, *R*^2^ = 0.09)				
Intercept	1.52	1.01	1.5	0.13
Δ wing disc loading	62.5	28.27	2.21	0.02
Relatedness between contenders	−10.4	5.64	−1.84	0.06
**Random effects**	Variance	SD		
Total number of flowers in the foraging patch	0.43	0.65	–	–
Foraging patch ID	0.73	0.85	–	–

The genetic relatedness between the hummingbird species measured by the genetic distance was within a range of 0.028 to 0.241 (Table S4). The most distantly related contending species were Green-breasted Mango and White-bellied Emerald (genetic distance = 0.241). The most closely related species were two species of the genus *Saucerottia* (Berylline Hummingbird and Azure-crowned Hummingbird; genetic distance = 0.028).

**Figure 3:**
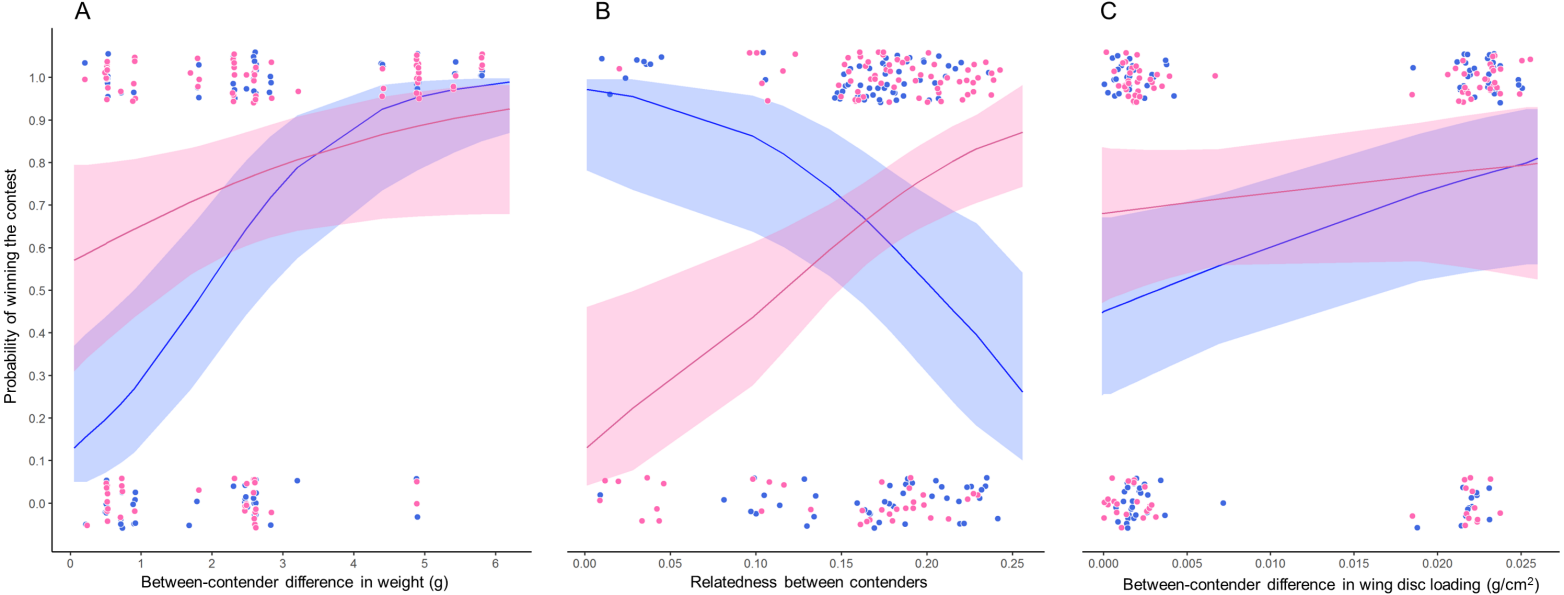
Probability of winning contests as function of trait difference between contenders. GLMM plots relating two binomial response variables (1) when winner is due to weight (pink dots) and (2) when winner is due to wing disc loading (blue dots) with (A) weight difference between contenders, (B) relatedness between contenders and (C) wing disc loading difference between contenders. Pink lines are the probability that the heaviest contender win contests and blue lines are the probability that the hummingbird with greatest wing disc loading win contests. These probabilities were estimated through GLMMs with binomial distribution. Pink and blue ribbons are the 95% confidence intervals of each model. Only the significant main predictor variables are plotted for each model. Model 1 pink dots and lines in A and B; Model 2 pink dots and lines in C; Model 3 blue dots and lines in A and B; Model 4 blue dots and lines in C. Dots were jittered to indicate sample sizes.

**Figure 4:**
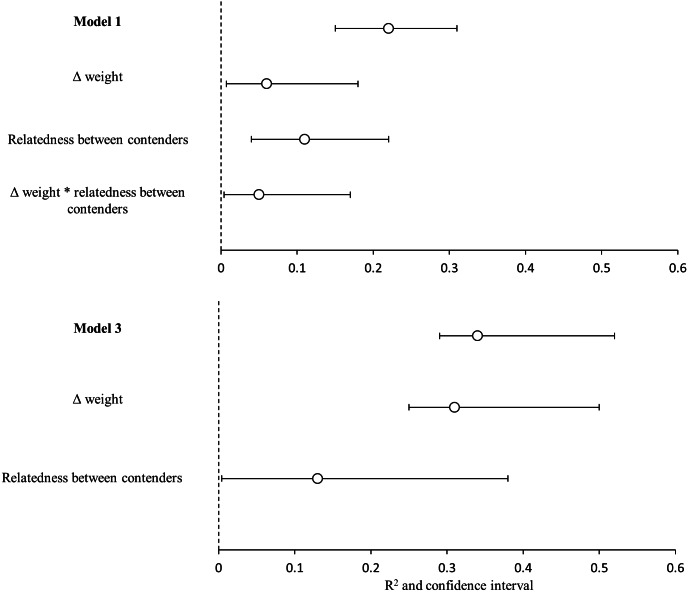
Variance explained by the GLMMs. Variance explained (*R*^2^) for the model 1(heaviest hummingbird wins (1) or loses (0) the contest ∼Δ weight * relatedness between contenders) and for model 3 (hummingbird with greatest wing disc loading wins (1) or loses (0) the contest ∼Δ weight + relatedness between contenders); and the contribution of each predictor variable (semi-partial *r*^2^). White dots are the mean value of *r*^2^ and black lines are the 95% confidence intervals. Variables with the symbol Δ indicate difference between contenders. The asterisks indicate the interactions between variables. Model *R*^2^ is not the sum of partial *r*^2^ values, due to the intercorrelations between the predictor variables.

The bests GLMMs were model 1 and model 3. Model 1 indicated that the probability that the heaviest hummingbird won contests was positively associated with the differences in weight and genetic relatedness between contenders (Table 4; Fig. 3). Model 3 indicated that the probability that the hummingbird with greatest wing disc loading won contests was positively associated with the differences in weight and negatively with the genetic relatedness between contenders (Table 4; Fig. 3). The *R*^2^ value for the full model 1 was 0.22 (95% CI [0.15–0.31]; Fig. 4). Among the main variables of model 1, the relatedness between contenders had the highest *r*^2^ (0.11). However, the rest of the variables of the model had a similar explanatory power (Fig. 4). On the other hand, the *R*^2^ value for the full model 3 was 0.34 (95% CI [0.29–0.52]; Fig. 4). The main variable of the model with higher explanatory power was the difference in weight between contenders (*r*^2^ = 0.31; Fig. 4). Model 4 was the only one in which the difference in wing disc loading was positively associated with the probability that the hummingbird with greatest wing disc loading won contests. However, this model had the lowest *R*^2^ = 0.09 (95% CI [0.01–0.23]; Fig. 4).

## Discussion

Most of our predictions were met. Our results indicate that during the flowering season of *P. padifolia* the hummingbirds in the CFS established dominance relationships to access their flowers. Both inferred dominance hierarchy indexes were positively correlated. As we expected a greater weight is the main contributor to dominance. Hummingbirds visited similar number of flowers regardless their weight or their dominance. The probability that the heaviest contender won contests was positively associated with the differences in weight and Tamura and Nei genetic relatedness between contenders. On the other hand, the probability that the contender with greatest wing disc loading won contests was positively associated with differences in weight and negatively with the genetic relatedness between contenders. However, these models only explained between 22% and 34% of the variation in the probability that the heaviest contender and the contender with greatest wing disc loading win the contests, respectively.

### Dominance hierarchy

The agonistic interaction network had 31% of the possible pairwise interactions. These results imply that not all hummingbird species participated in contests to ensure their access to floral nectar. Hummingbirds can modify their foraging behaviors according to resources availability, abundance and identity of competitors or resource preferences (Feinsinger & Colwell, 1978). In the CFS the Azure-crowned Hummingbird, Wedge-tailed Sabrewing, Rivoli’s Hummingbird and Berylline Hummingbird were the most frequently seen visiting the flowers and they were also the species that participated most frequently in contests and against more species (*i.e.*, highest degree = 6 pairwise interactions). These species were frequently observed defending floral patches or even were nectar thieves in other defended patches (Ornelas et al., 2004a). Conversely, Bumblebee Hummingbird almost completely avoid agonistic interactions to access the nectar of flowers (*e.g.*, lowest degree = 1 pairwise interaction). However, none of the species had absolute dominance over the rest of the hummingbirds in the assemblage. This highly variable hierarchy (wide 95% confidence intervals in ranks measured by Elo-rating) implies that the dominance relationships between species are weak. In a complete linear hierarchy, there is one dominant species who dominates all the other species, a second who dominates all but the most dominant species, so continue down to the most subordinate species who dominates no one (Chase & Seitz, 2011). In both inferred dominance hierarchy indexes the most dominant hummingbird it was different (David’s score = Wedge-tailed Sabrewing; Elo-rating = Rivoli’s Hummingbird). However, both species lost between 29–30% of their contests respectively (Table 2). Interactions between hummingbird species in the CFS have a hierarchical structure. These hierarchical interactions are relatively stable and allow predicting the outcome of a contest between pairwise interactions. Nevertheless, when all species are included to infer the dominance hierarchy, it becomes weak and loses its predictive capacity to assign ranks to species. This can be promoted by differences between the foraging strategies of hummingbird species in the CFS and their ability to modify their foraging behavior can promote their coexistence even when all the species need to ensure the access to the same floral resource. Nevertheless, the wide range of foraging strategies and the plasticity of hummingbird behavior can generate a high degree of uncertainty when inferring the species dominance position within a hierarchy, mainly for non-territorial species. Considering that, it is more informative to use methods to infer the dominance hierarchy that estimate the uncertainty of the position of each species within the hierarchy (*e.g.*, randomized Elo-rating; Sánchez-Tójar, Schroeder & Farine, 2018). These methods can more clearly reflect the dynamics in the dominance relationships between species.

### Contributors to high dominance values

Multiple linear regression analysis suggested that greater weight and wing disc loading contribute to dominance measured by Elo-rating and David’s score, but wing disc loading explained less than weight. While we found that wing disc loading was associated with both hierarchies, other studies failed to find a relationship with David’s score hierarchy (Altshuler, Stiles & Dudley, 2004; López-Segoviano, Bribiesca & Arizmendi, 2017). The lack or small relationship between the wing disc loading and dominance can be explained for the evolution of disproportionate increases in muscle capacity and wing size in larger species that generate lower wing loading values (Skandalis et al., 2017; Dakin et al., 2018). In this study, the wing disc loading estimate for the non-territorial Bumblebee Hummingbird (0.038 g cm^−2^) was similar to that of the territorial Rivoli’s Hummingbird (0.032 g cm^−2^). This minimal difference in hummingbirds with opposite foraging behaviors supports that wing disc loading is a poor predictor of dominance behavior (Altshuler, Stiles & Dudley, 2004; Sargent, Groom & Rico-Guevara, 2021). In future research, other predictors of flight performance should be calculated experimentally and included in the study of dominance dynamics in hummingbird assemblages (*e.g.*, Burst power; Segre et al., 2015; Sargent, Groom & Rico-Guevara, 2021). While weight is associated with dominance in hummingbirds (Rodríguez-Flores & Arizmendi, 2016; Márquez-Luna et al., 2018; Bribiesca et al., 2019), it is not overwhelmingly important and heaviest species do not always win. Indeed, other factors could influence dominance, for example the availability of resources, the preference for particular resources, the hummingbird previous experience, and even its body condition associated to their hunger level. In fact, this pattern has been reported in other places, where the heaviest species dominated the rest of the species in the hummingbird assemblages (Dearborn, 1998; Justino, Maryama & Oliveira, 2012). However, species weight explained 0.61 and 0.76 of the variance in dominance measured by Elo-rating and David’s score respectively and so the heaviest species are not always winners.

While weight is strongly associated with dominance (López-Segoviano, Bribiesca & Arizmendi, 2017; Francis et al., 2018), hummingbird weight can be quite variable intraspecifically. For example, weight gain can be 0.25–0.50 g day^−1^ in stopover sites of latitudinally migratory hummingbirds (*e.g.*, Rufous Hummingbird, *Selasphorus rufus*; Carpenter, Paton & Hixon, 1983). Furthermore, in controlled conditions Anna’s Hummingbird (*Calypte anna*) and Costa’s Hummingbird (*Calypte costae*) can increase their weight up to 0.69 and 0.53 g day^−1^ respectively. However, during the night hummingbirds have a constant weight loss of between 0.042–0.053 g hour^−1^ (Powers, 1991). It is necessary to conduct studies on this topic, considering that influences competitive ability (Dakin et al., 2018). Additionally, it is important to consider that some morphological traits are quite variable within species. This morphological variability can be the result of the different conditions and resources to which populations have been exposed over time (*e.g.*, Azure-crowned Hummingbird, Rodríguez-Gómez, Gutiérrez-Rodríguez & Ornelas, 2013; Rivoli’s Hummingbird, Zamudio-Beltrán et al., 2020). These differences between populations could influence the behavior of the same species throughout its geographic distribution.

Another possible behavioral response is that hummingbirds use the body size of the contender as a visual cue to determine the costs and benefits of an agonistic contest. This could explain the ineffective territorial defense of Azure-crowned Hummingbird as it lost most of its contests against contenders with a larger body size. It has been reported that White-eared Hummingbird (*Basilinna leucotis*) use the length of their superciliums as a badge of dominance. Males with larger superciliums had enhanced access to resources than males with smaller superciliums (González-García et al., 2018). The hummingbirds could use the body size of their contenders to regulate their response to competitor. This response based on the contender body size could avoid the investment of energy in fights that the hummingbird could lose, reduce the risk of injury or increase the aggressiveness of the behavioral response when both contenders are of similar size. However, further research is required to assess whether this occurs in interspecific contests.

### Number of flowers visited

Hummingbirds visited similar number of flowers regardless their weight or their dominance. The number of visited flowers per foraging bout increases slightly with the species dominance. Despite this increase, our results suggest that all hummingbird species had variable foraging bouts in which they were able to visit many or few flowers regardless of their dominance. Other studies have documented segregation in the used resources between the hummingbird species, where the heaviest species monopolize the access to the best nectar resources (López-Segoviano, Bribiesca & Arizmendi, 2017). Nevertheless, in our study site the *P. padifolia* flowers were the most abundant resource and all hummingbird species used this resource. The heaviest species tended to be dominant over the smaller. Additionally, within a species the heaviest individuals usually performed slower accelerations than lighter conspecifics (Dakin et al., 2018). This is because; searching for resources implies a greater energy invests for larger individuals. This energy demand is met by visiting more flowers per foraging bout within territories or in foraging routes (*e.g.*, high reward trapliner; Feinsinger & Colwell, 1978). Dominant hummingbirds tend to establish and defend foraging patches that guarantees them nectar access without investing energy in searching for it. Likewise, in this foraging strategy the owner of the floral patch invest time and energy in the territorial defense (through vocalizations or intruder chases), but the energy within the defended patch covers the costs of the territorial defense (Kodric-Brown & Brown, 1978). However, it is common for territorial hummingbirds to leave their territories when expelling intruders or to feed on nearby flowers (Justino, Maryama & Oliveira, 2012). These absences are taken advantage by other hummingbirds (non-territorial or territorial parasites) to feed on flowers within the defended patch. The non-territorial hummingbirds can avoid the energetic costs associated with the territorial defense and can also cover their energy needs foraging on non-defended flowers or to taking advantage of the periods of absence of territorial hummingbirds. Nevertheless, when the chases escalate into physical contact, intruders are at increased risk of injury or having their feathers plucked by territorial hummingbirds (Rico-Guevara & Araya-Salas, 2015). Besides the injury risk, the feathers loss can generate an energetic cost, because the loser would have to invest energy in the keratin synthesis to replace their feathers (King & Murphy, 1990).

The behavioral plasticity of hummingbirds allows them to maximize their energy intake even establishing foraging routes (traplining foraging) in small spatial scale like a bush or even within a floral patch (Tello-Ramos, Hurly & Healy, 2015). The dynamic foraging behavior of hummingbirds can benefit the reproductive success of *P. padifolia*. The slight difference in nectar volume and sugar concentration produced by L-morph and S-morph promotes the use of both floral types by hummingbirds, transferring pollen loads between them (Ornelas et al., 2004a). Considering this, the territorial behavior could limit the pollen flow between morphs, but the transfer of pollen can be increased when the owner of the territory leaves it to feed on nearby flowers; or when the non-territorial hummingbirds (intruders) manage to feed on flowers within defended floral patches (Ornelas et al., 2004a; Justino, Maryama & Oliveira, 2012). These results also suggest that the hummingbird dominance was weak and did not have a major effect on resource use.

### Difference between contenders

Our results indicate that when the weight difference between contenders was less than 2 grams, both contenders (*i.e.*, the biggest and the smaller and, the contender with greatest and lowest wing disc loading) had similar probabilities to win a contest (Fig. 3A). Our results support the prediction that fluctuations in body mass of between 10 and 20% will affect the competitive abilities of hummingbirds (Dakin et al., 2018). The greater the difference in weight between contenders, the greater the probability that the heaviest contender will win the contest. This pattern was similar to that reported in several taxa (*e.g.*, Poeciliid Fish, *Xiphophorus helleri*, Beaugrand & Cotnoir, 1996; Green Anole, *Anolis carolinensis*, Bush et al., 2016; Shrews, Rychlik & Zwolak, 2006; Domestic Mouse, *Mus musculus*, Varholick et al., 2018). These studies suggest that the effect of the greater weight is diluted when the contenders have similar weight and then the order in the dominance hierarchy becomes unstable or is explained by other morphological traits (*e.g.*, greater muscle capacity; Dakin et al., 2018) or by the previous experience of the contenders (Beaugrand & Cotnoir, 1996).

The genetic relatedness between contenders showed two patterns. (1) The lower relatedness between contenders, the greater is the probability that the heaviest contender will win the contest. (2) The greater relatedness between contenders, the greater is the probability that the contender with the greater wing disc loading will win the contest. The genetic distance between the hummingbird species in our assemblage was within a range of 0.028 to 0.241. We did not record contests between the most distantly related species (Green-breasted Mango and White-bellied Emerald); and there were few contests among the most closely related species (Beylline Hummingbird and Azure-crowned Hummingbird). The genetic distance between these species was only 0.028. Eight out of the nine contests were won by the smaller contender (Berylline Hummingbird) and only one contest was won by the heaviest contender (Azure-crowned Hummingbird). The contests won by the smallest contender did not show a clear pattern, since they occurred among closely and distantly related contenders. The relatedness between contenders can reflect the morphological similarity between species. This implies that closely related species could have similar morphological traits and maneuvering styles (Skandalis et al., 2017; Dakin et al., 2018). The opposite pattern could occur between distantly related contenders. This implies that there will be greater morphological and physiological variability that can generate a behavioral divergence between distantly related species (Dakin et al., 2018). This divergence can be exploited by hummingbird species with lower wing disc loading to win contests against competitors with greater wing disc loading. However, in future research it would be interesting to evaluate these morphometric traits through phylogenetically controlled analyses to evaluate what of these variables are adaptive to defend foraging territories.

When morphology of contenders is similar, other factors may become more important in determining the outcome of the contest (Chase & Seitz, 2011). For example, the experience of having won or lost a contest or the previous residence in a territory. Observational learning processes related with their experience have been documented in hummingbirds. For example, when the exposition time to new resources (*i.e.*, feeders with different sugar concentration) increases, the Lucifer Sheartail (*Calothorax lucifer*) females increase the frequency of agonistic contests around the highest sugar concentration feeders (Márquez-Luna, Lara & Ortiz-Pulido, 2017). Likewise, it has been reported that the previous territory residence has an effect in the agonistic contest outcome in White-eared Hummingbird (Mendiola-Islas et al., 2016).

### Limitations

Our GLMMs explained only the 22% of the variation in the probability that the heaviest contender won contests and the 34% of the variation in the probability that the contender with greatest wing disc loading won contests. Most of this variation was explained by genetic relatedness and difference in weight between contenders (semi-partial *r*^2^ = 0.11 and 0.31 respectively). In addition to our other results this reflects the weak dominance relationships between the species competing for the access to *P. padifolia* flowers. The heaviest species did not win all its contests. This weak dominance hierarchy can be promoted by the different foraging strategies and the behavior plasticity in hummingbirds. Also this foraging behavior variability can promote the coexistence of hummingbird species that need to ensure the access to the same floral resource. Although weight has been reported to be a good indicator of dominance in hummingbirds the rest of the variability in dominance could be explained by other variables involved in determining the outcome of the contests. Sargent, Groom & Rico-Guevara (2021) have suggested that the use of the burst power can be an indicator of interspecific dominance. The burst power is a measure of muscular capacity (measured as the maximum mass of beads that the bird can lift in vertical flight; Dakin et al., 2018; Sargent, Groom & Rico-Guevara, 2021). The territorial hummingbirds consistently develop more burst power than non-territorial. The enhanced maneuverability (*i.e.*, the ability to change the speed and direction of flight) in hummingbirds is directly related to an increase in muscular capacity (burst power) rather than weight (Dakin et al., 2018; Sargent, Groom & Rico-Guevara, 2021). However, currently the burst power of few species has been estimated (Segre et al., 2015; Dakin et al., 2018). The further research of interspecific dominance in hummingbirds must be focused on the estimation of burst power data. The more data are estimated for different assemblages of hummingbirds, we can obtain more accurate models that help us understand the dynamics of hummingbird dominance in different ecological contexts.

## Conclusions

Weight is the major contributor to high dominance values within dominance hierarchies. All the hummingbird species present in the assemblage of the CFS have developed behavioral mechanisms that allow them to access nectar even without participating in agonistic contests or even losing them. The differences in weight and genetic relatedness between contenders have effects on the probability that a species will win an agonistic contest. This promotes that the dominance hierarchy is highly variable and the dominance relationships between species tend to be weak. Future studies should include (1) the temporal variability of the weight and (2) the burst power of the hummingbirds to evaluate its effects on the dynamics of dominance hierarchies in hummingbird assemblages.

## Supplemental Information

10.7717/peerj.13331/supp-1 Table S1 Hummingbird species morphological traits The data were collected in 1999 in the CFS during the flowering season of Palicourea padifolia. Are indicated the mean and the confidence intervals in parentheses, *n* = 8 males and 7 females for all species. Species are order by weight.

10.7717/peerj.13331/supp-2 Figure S1 Relationship between morphological traits of hummingbirds Relationship between weight and wing length of hummingbirds in the CFS. Abbreviations as in Fig. 1.

10.7717/peerj.13331/supp-3 Table S2 ND2 sequence alignment for the hummingbird species registered in the Cloud Forest Sanctuary

10.7717/peerj.13331/supp-4 Table S3 Interactions of hummingbirds in the CFS Total visits to Palicoura padifolia flowers, number of foraging bouts per hummingbird species and interactions registered in the Cloud Forest Sanctuary.

10.7717/peerj.13331/supp-5 Figure S2 Relationship between dominance hierarchies Black dots indicate the position of each species within dominance hierarchies. Abbreviations as in Fig. 1.

10.7717/peerj.13331/supp-6 Table S4 Genetic distance between the hummingbird species in the CFS The values below the diagonal represent the genetic distance estimated through the Tamura-Nei model using ND2 gene sequences. Mex = Mexican Violetear, Colibri thalassinus; Gre = Green-breasted Mango, Anthracothorax prevostii; Riv = Rivoli’s Hummingbird, Eugenes fulgens; Ame = Amethyst-throated Mountain-gem, Lampornis amethystinus; Bum = Bumblebee Hummingbird, Selasphorus heloisa; Wed = Wedge-tailed Sabrewing, Pampa curvipennis; Lon = Long-tailed Sabrewing, P. excellens; Azu = Azure-crowned Hummingbird, Saucerottia cyanocephala; Ber = Berylline Hummingbird, S. beryllina; Buf = Buff-bellied Hummingbird, Amazilia yucatanensis and Whi = White-bellied Emerald, Chlorestes candida

## Additional Information and Declarations

### Field Study Permissions

The following information was supplied relating to field study approvals (*i.e.*, approving body and any reference numbers): Field work was approved by the administation of the Francisco Javier Clavijero botanical garden.

### Data Availability

The following information was supplied regarding data availability: The data supporting this study are available in the Open Science Framework: Luna, Ubaldo M. 2022. “Data Repository.” OSF. March 10 osf.io/hsygt.
