# Supplementary figures and images for: Genetic relatedness and morphology as drivers of interspecific dominance hierarchy in hummingbirds
Source: PeerJ. 2022 Apr 20;10:e13331. doi: 10.7717/peerj.13331 (PMC9034699; doi:10.7717/peerj.13331)

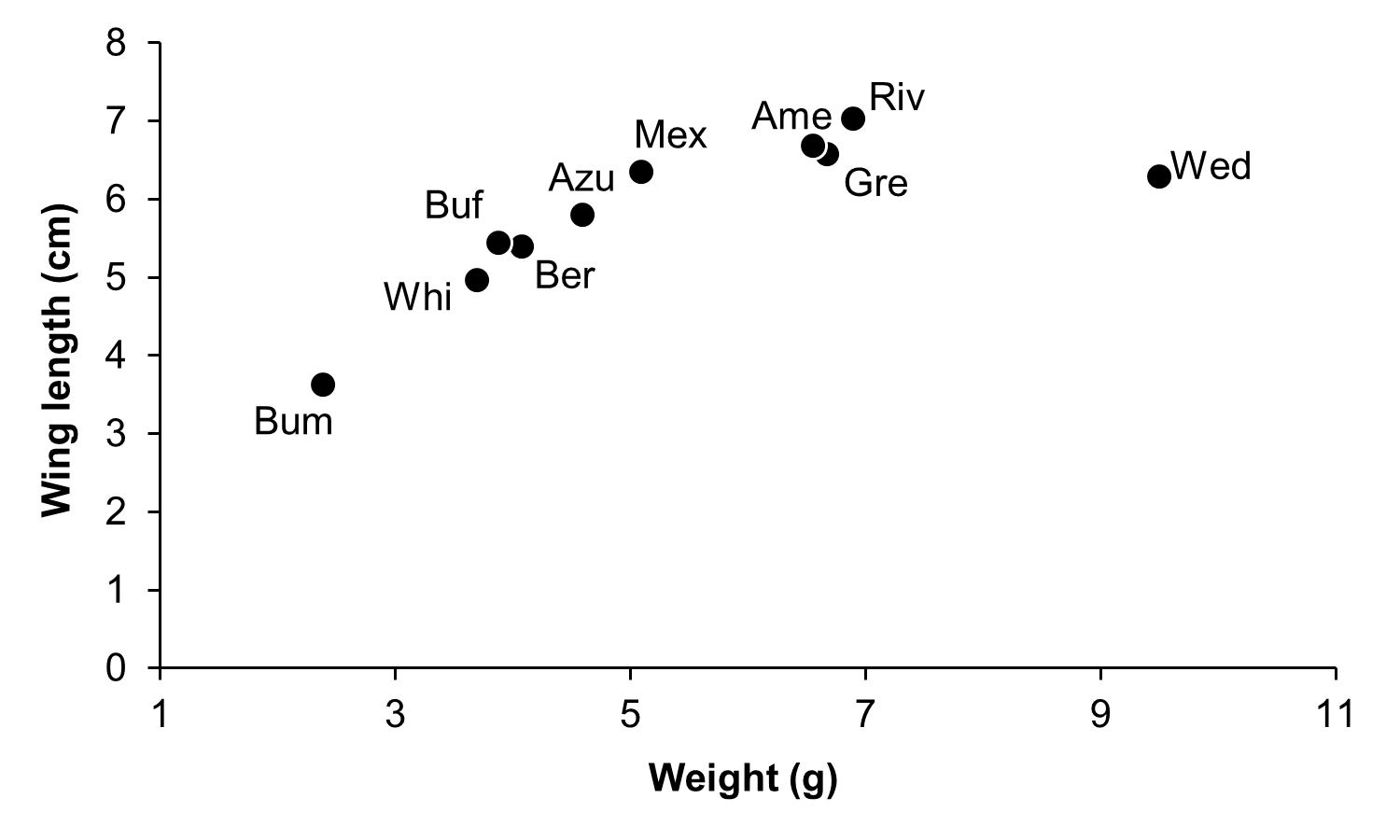

Supplement: Figure S1 — Relationship between weight and wing length of hummingbirds in the CFS. Abbreviations as in Fig. 1. [file peerj-10-13331-s002.png]

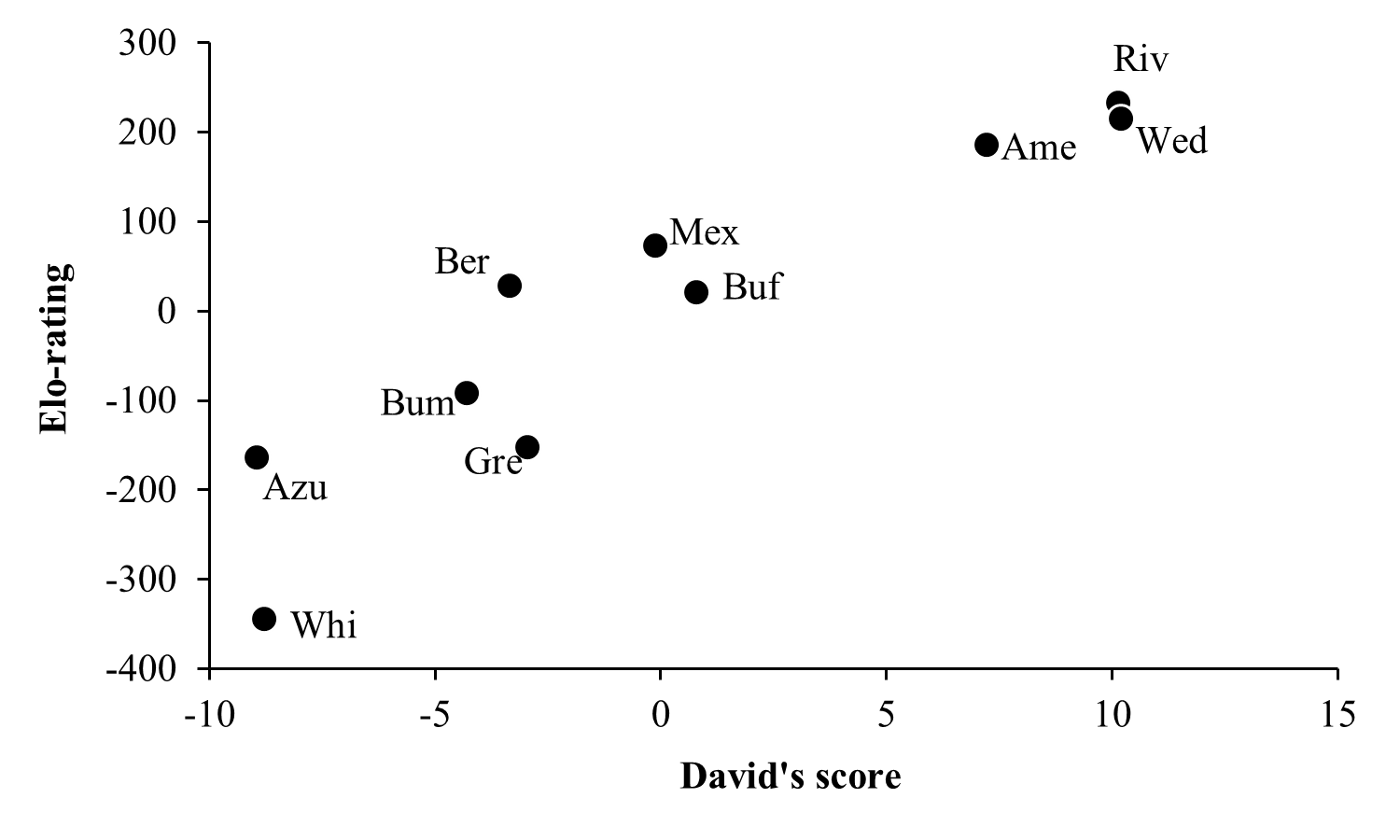

Supplement: Figure S2 — Black dots indicate the position of each species within dominance hierarchies. Abbreviations as in Fig. 1. [file peerj-10-13331-s005.png]
