# Supplementary material for: Genetic relatedness and morphology as drivers of interspecific dominance hierarchy in hummingbirds
Source: PeerJ. 2022 Apr 20;10:e13331. doi: 10.7717/peerj.13331 (PMC9034699; doi:10.7717/peerj.13331)
Supplement: Table S2 [file peerj-10-13331-s003.pdf]

Table S2. ND2 sequence alignment for the hummingbird species registered in the Cloud Forest Sanctuary.

Species GenBank accession number ND2 sequence (1041 bp)

Chaetura pelagica (outgroup) MH719133.1
ATGAACCCTTACGCTAAACTAATCTTCACCATAAGCCTACTCCTAGGAACAACCATAACAATCTCTAGCAGCC
ACTGAATCCTAGCCTGGACAGGACTAGAAATCAACACCCTAGCCATCATCCCACTCATCGCAAAACACCACCA
CCCACGAGCCATCGAAGCCGCAATCAAATATTTCCTAGTCCAAGCAGCCGCCTCAACACTACTACTATTCTCA
AGCACAATTAATGCCTGGTCCTCCGGACAATGGGACATCACACAACTAAGCCACCCCATCTCATGTGCCCTAC
TAACAACAGCGATTGCAATAAAACTCGGGCTAGTCCCATTCCACTTCTGATTCCCAGAAGTCCTCCAAGGATC
CTCATTAACAACCGCAATACTCCTATCAACAATAATAAAATTCCCCCCAATCACCATCCTTCTCATAACAGCC
CACTCACTAAACCCAGCCCTCCTTACCACCATGGCAGTTTCTTCAGCCGCCCTTGGAGGCTGAATAGGGCTTA
ACCAAACACAAACTCGAAAAATCCTAGCTTTCTCATCTATCTCTCACCTCGGATGAATAGCTATCATCCTTAT
TTACAACCCCAAACTAACCCTACTAACCTTCTACGTATATTGCCTTATAACCATCACTGTATTCCTCACCCTA
AACACAACAAAAACTCTTAAACTATCTACAATAATAACTTCCTGAACAAAAACCCCAACACTAAATGCAGCCT
TAATACTAACTCTCCTATCCTTAGCCGGACTCCCACCTCTAACAGGATTCTTACCTAAATGACTCATCACTCA
AGAACTTACCAAACAAGAAATAACTTCAACAGCCACAATCATCACACTCCTCTCCCTTCTAGGCCTATTCTTC
TACCTCCGCCTCGCATACTACTCAACAATTACACTTCCTCCCAACCCCACAAACCACATAAAACAGTGACATG
TCTACAAACTAACAAATACCCTCATTCCCATCCTCACAACACTCTCTACCCTGCTCCTCCCCCTCTCCCCCAT
AATCCTTGCCACTATTTAG
Atthis heloisa KX855345.1
ATGAACCCCTACGCTAAACTAACTTTCTCTTTGAGCCTTATATTAGGGACAACCATCACCATTTCAAGTAGCC
ATTGAATGTCGGCCTGGGCCGGACTTGAGATCAACACCCTGGCCATCATCCCGCTTATCTCAAAGTCCCACCA
CCCCCGAGCCATTGAAGCAGCGATCAAATATTTCCTCGTCCAAGCTGCTGCCTCTACCCTACTGCTATTCTCA
AGCATAATTAATGCCTGACACACAGGACAATGAGACATCACTCAACTAAACCACCCCACATCATCCCTATTAC
TAACCACAGCGATTGCAATAAAACTTGGCTTGGTACCCTTTCACTTCTGATTCCCCGAAGTCCTGCAAGGCTC
CCCCTTAACAACAGCCATACTCTTATCAACAGTAATAAAATTCCCTCCAATGACGATCCTTTTCCTCACATCC
CACTCCCTAAACCCAACCCTACTCACTACCATAGCACTTGCTTCAGCCGCTCTAGGGGGGTGAATAGGACTAA
ATCAAACACAGACTCGAAAAATCCTAGCCTTCTCATCAATCTCTCATTTAGGGTGAATAACCATCATCCTCAT
CTACAACCCAAAACTAACACTAGTAACCTTCTACCTTTATTGCATCATAACCGGTGCCATCTTCCTAACTTTA
AATACCACAAAATCCCTTAAACTCTCAACGATAATAACCTCCTGAACAAAAACTCCCGTACTTAACACCACAT
TAATAATAACACTACTCTCACTAGCAGGATTACCCCCACTAACAGGATTCCTGCCCAAATGACTCATCATCCA
AGAACTCTCCAAGCAAGAAATAGCCACCTCCGCTACAATTATCGCCATCCTATCCCTACTGGGCCTATTCTTC
TATCTCCGACTAGCCTACTACTCAACAATCACTCTTCCGCCTAACCCCACAAACCATATAAAACAATGACACA
CTAATAAACGCACAAGCGTCCTAATCGCTACACTCTCCTCCTTGTCAACCCTCTTACTCCCACTATCCCCAAT
AGTCCTCGCCTCCATCTAA
Lampornis amethystinus KJ602255.1
ATGAACCCCTACGCTAAATTAACATTCCTTATAAGCCTTATGCTAGGAACAACCATCACTGCTTCAAGTAATC
ACTGAGTGTCAGCCTGAGCCGGACTTGAAATCAACACCCTAGCCATCATTCCCCTAATTTCTAAGTCCCACCA
CCCCCGAGCCATTGAAGCAGCAATCAAATATTTCCTTGTCCAAGCTGCTGCCTCCACTCTTCTATTATTCTCA
AGCATGATTAATGCTTGACACACAGGACAATGAGACATCACTCAACTAAACCACCCCACATCATCCCTACTAC
TAACCACAGCAATTGCAATAAAACTCGGCCTTGTGCCCTTCCATTTCTGATTCCCAGAAGTCCTGCAAGGCTC
CCCCCTAACCACAGCTATACTTCTATCTACAGTAATAAAATTCCCCCCAGTCACCATTCTTTTCCTCACATCT
CACTCCCTAAACCCAACTCTACTTACCATAATAGCCCTAGCCTCCGCCGCATTAGGGGGGTGAATAGGACTAA
ACCAAACACAAATTCGTAAAGTCCTAGCCTTCTCATCAATCTCACACCTAGGATGAATGGCCATTATCCTTAT
CTACAACCCAAAACTAACATTAATAACCTTCTACCTATACTGTGTCATAACCAGCGCCATCTTCCTAACCCTA
AACACCACAAAATCCCTCAAACTATCAACAATAATGACCTCCTGGACAAAAACTCCTGTACTCAACACCGCAT
TAATAATAACACTACTCTCATTGGCAGGCTTACCCCCACTTACAGGATTCCTACCCAAATGACTTGTTATCCA
AGAACTCTCCAAACAAGAAATATCTTCTTCTGCCACAGTTATTGCCATCTTGTCCTTATTGGGTTTATTTTTC
TACCTCCGTCTAGCCTACTACTCAACCATCACTCTTCCACCAAACCCCACAAACCACATAAAACAATGATACA
CTTATAAACCCACAAGCAGCCTAATCGCCACGCTCTCCTCCCTATCAGCCCTCTTACTCCCACTGTCCCCAAT
AATCCTCGCCTCCATCTAA

Pampa excellens* KJ602200.1
ATGAACCCGTACGCTAAACTAACATTCTCCACAAGCCTTATGCTGGGAACAACCATCACCATCTCTAGCAGCC
ATTGAATAGCCGCCTGAGCCGGACTTGAAATCAACACCTTAGCCATCATCCCCCTTATCTCAAAATCCCACCA
CCCTCGGGCCATCGAAGCAGCAATTAAATACTTTCTAGTGCAAGCTGCTGCCTCTACTTTACTTCTATTCTCA
AGCATAATTAATGCCTGACACACAGGACAGTGAGACATCACTCAACTAAATCACCCCACATCATCCCTCCTAC
TCACTACAGCTATCGCGATAAAACTCGGCCTAGTGCCCTTCCACTTTTGATTCCCGGAAGTCCTACAAGGCTC
CCCCCTAATCACTGCCATGCTTCTATCAACAGTAATAAAATTTCCCCCAATCACCATCCTCTTCCTCACATCC
CACTCACTAAACCCCACCCTACTCACCACAATAGCAATCGCTTCAGCCACATTGGGAGGATGAATGGGACTGA
AYCAAACACAAACCCGAAAARTCTTAGCTTTCTCATCAATCTCCCACCTAGGATGAATAACCATCATCCTTAT
CTACAACCCAGAACTAACATTAATAACCTTCTACCTTTACTGCATTATAACCGGCGCTATCTTCCTGACACTC
AATACTACAAAATCTCTTAAACTATCAATAATAATAACTTCCTGAACAAAAACCCCTGTACTTAACACAACAT
TAATAATAACATTACTCTCACTAGCAGGCCTACCCCCACTAACAGGTTTCTTGCCTAAATGACTTATCATTCA
AGAACTTTCCAAGCAGGAAATATCCACCTCCGCCGTAGCCATCGCTATCCTATCCTTACTCGGCCTATTTTTC
TACTTACGCCTAGCCTACTACTCAACAATCACCCTACCACCAAGCCCTACAAACCATATAAAACAATGATACA
CTAACAAACCCACAAACACCTTAATTGCCCTACTCTCTTCTTTATCAGCCCTTTTACTCCCACTATCCCCAAT
AATCCTCGCCTCCATCTAA
Amazilia yucatanensis KJ602180.1
ATGAACCCTTACGCCAAACTAACATTCTCCATAAGTCTTATGTTAGGAACAACCATCACCATTTCTAGCAACC
ACTGAATGTCAGCCTGAGCCGGACTCGAAATCAACACCCTAGCCATTATCCCCCTTATTTCAAAATCCCACCA
CCCCCGAGCCATTGAAGCAGCAATCAAATACTTCCTCGTACAAGCCGCTGCCTCCACCTTACTATTATTCTCA
AGCATAGTCAATGCTTGACACACAGGACAATGAGATATCACGCAACTAAACCACCCTACATCATCCCTGCTAC
TAACTGCAGCAATTGCAATAAAACTTGGCCTAGTACCCTTCCACTTCTGATTTCCAGAGGTCTTACAAGGCTC
CCCCTTAATTACAGCCATACTTTTATCAACAGTAATAAAATTTCCCCCTATCACTATCTTCTTCCTCACATCC
CACTCATTAAACCCTACCCTACTCACCACAATAGCAATTGCCTCAGCTGCCCTAGGAGGATGAATGGGACTAA
ATCAAACCCAAACCCGAAAAATTCTAGCCTTCTCATCAATCTCCCACCTAGGATGGATAACCATCACCCTCAT
CTACAATCCAGAACTAACATTAATAACCTTCTACCTCTATTGTGCTATAACCAGTGCTATCTTCTTAACCCTT
AACACCACAAAGTCTCTCAAACTATCAATGATAATAACATCTTGAACAAAAGCCCCTGTACTTAACACAACTC
TAATAATAGCACTTCTCTCACTAGCAGGTCTCCCCCCACTAACAGGCTTCCTGCCAAAATGGCTTATTATCCA
AGAACTCTCTAAACAAGAAATAGCCGCCTCCGCCACAATCATCGCCGTCCTATCCCTACTAGGCCTATTCTTC
TACCTTCGCTTAGCCTACTACTCAACAATCACCCTACCCCCAAGCCCCACGAACCACATAAAACAATGATATA
TTAATAAACCCACAAACACCTTAATCGCCACACTTTCCTCCATATCAACCCTCCTGCTCCCACTATCTCCGAT
AATCCTTGCCTCCATCTAA
Saucerottia cyanocephala KJ602167.1
ATGAACCCTTACGCCAAACTAACATTCCTTATAAGTCTCATGCTAGGAACAGCCATCACTATCTCTAGCAATC
ACTGAATGTCAGCCTGAGCCGGACTCGAAATCAACACCCTAGCCATCATCCCCCTTATCTCAAAATCCCACCA
TCCCCGAGCTATCGAAGCAGCAATCAAATACTTCCTCGTCCAAGCCGCTGCTTCCACCCTGCTATTATTCGCA
AGCATAATCAATGCCTGAAACACAGGACAATGAGATATTACCCAACTAAACCACCCCACATCATCCATGCTAC
TAACTACAGCAATTGCAATAAAACTAGGCCTAGTACCCTTCCACTTCTGATTCCCAGAGGTCTTGCAAGGCTC
CCCCTTAATTACAGCCATACTTTTATCAACAGTAATAAAATTCCCCCCAATCACCATCTTCTTCCTCACATCC
CACTCACTAAACTCCACCCTACTCACCACAATAGCAATCGCTTCAGCCGCCCTAGGAGGATGAATAGGGCTAA
ATCAAACCCAAACCCGAAAAATCCTAGCCTTCTCATCAATCTCCCACCTAGGATGAATAGCCATCATCCTCAT
TTATAACCCTAAGCTAACACTAATAACCTTCTACCTTTATTGCACTATAACCGGTGCTATCTTCTTAGCCCTT
AACACTACAAAATCCCTTAAACTATCAATAATAATAACATCCTGAACAAAAGCTCCTGTACTCAACACAACCC
TAATAATAGCACTACTCTCATTAGCAGGCCTGCCCCCACTGACAGGCTTCCTACCAAAGTGACTTATTATCCA
AGAGCTCTCTAAGCAAGAAATAGCCCCCTCCGCCACAATCATCGCCGTCCTATCCCTGCTAGGTTTATTCTTN
TACCTCCGCCTAGCCTACTACTCAACAATCACCCTACCACCTAGCCCCACAAACCACATAAAACAATGATACA
CTAATAAACCCACAAACACCATAATNGCCGCACTTTCCTCCATATCAACCCTCCTACTTCCACTATCCCCCAT
AATCCTTGCCTCCATCTAA
Chlorestes candida KJ602164.1
ATGAACCCTTACGCCAAACTAACATTCTCCGTAAGCCTCATGCTGGGAACAATCATCACCATTTCTAGCAATC
ACTGAATATCAGCCTGAGCTGGACTAGAAATCAACACTCTAGCCATCATCCCCCTTATTTCAAAATCTCACCA
CCCCCGAGCCATTGAAGCAGCAATCAAGTACTTCCTCGTACAAGCCGCTGCCTCCACCTTACTACTATTCTCA
AGCATAATCAATGCTTGACATACAGGGCAATGAGACATTACACAACTAAACCATCCCACATCATCCCTTTTGC
TAACTACAGCAATTGCAATAAAACTTGGATTAGTGCCCTTCCACTTCTGATTTCCAGAGGTCTTACAAGGCTC

CCCATTAATTACAGCCATACTTTTATCAACAGTGATAAAATTTCCCCCAATCACCATCCTCTTCCTCACATCC
CAATCACTAAACCCCGCCCTACTCACCACAATAGCAACCGCTTCAGCTGCCCTAGGGGGGTGAATAGGACTAA
ACCAAACCCAAACCCGAAAAATCCTAGCCTTCTCATCAATTTCCCACCTAGGATGAATAACCATCGTCCTTAT
CTACAATCCAGAACTAACATTAATAACCTTCTACCTCTACTGCTCTATAACCGGTGCTATCTTCTTAACCCTT
AACACTACAAAATCCCTCAAACTATCAATAATAATAACATCTTGAACAAAAGCTCCTGTACTTAACACAACCT
TAATAGTTGTTCTACTATCATTAGCAGGCCTACCCCCTCTAACAGGCTTCCTGCCAAAATGACTTATCATCCA
AGAACTCTCTAAACAAGAAATAGCCACCTCCGCCACAATCATTGCCATCCTATCCCTGCTAGGCTTATTCTTC
TACCTTCGTCTAGCCTACCACTCAACAATCACCCTACCACCAAGCCCTACGAACCACATAAAACAATGATATA
CCAATAAACCCACAAACACCTTAATCGCCACACTCTCCTCCACGTCAATCCTCTTACTCCCACTATCCCCCAT
AATCCTTGCCTCCATCTAA
Saucerottia beryllina KJ602161.1
ATGAACCCTTACGCCAAACTAACATTCCTTATAAGTCTCATGCTAGGAACAGCCATCACCATCTCTAGCAACC
ACTGAATATCAGCCTGAGCCGGACTCGAAATCAACACCCTAGCCATCATCCCCCTTATCTCAAAATCCCACCA
TCCCCGAGCCATTGAAGCAGCAATCAAGTACTTCCTCGTCCAAGCCGCTGCTTCTACCCTGCTATTATTCGCA
AGCATAATCAATGCCTGAAACACAGGACAATGAGACATTACCCAACTAAACCACCCTATATCATCCATGCTTC
TAACTACAGCAATTGCAATAAAACTCGGCTTAGTACCCTTCCACTTCTGATTCCCAGAGGTCTTGCAAGGCTC
CCCCTTAATTACAGCCATACTTTTATCAACAGTAATAAAATTCCCCCCAATCACCATCTTCTTCCTCACATCC
CACTCACTAAACTCCACCCTACTAACCACAATAGCAATCGCTTCAGCCGCCCTAGGAGGATGAATAGGGCTAA
ATCAAACCCAAACCCGAAAAATCCTAGCCTTCTCATCAATCTCCCACCTAGGATGAATAGCCATCATCCTCAT
TTATAATCCTAAGCTAACACTAATAACCTTCTACCTTTATTGCACTATAACCGGTGCTATCTTCTTAGCCCTT
AACACTACAAAGTCCCTTAAACTATCAATAATAATAACATCCTGAACAAAAGCCCCTGTACTCAACACAACCC
TAGTAATAGCACTACTCTCATTAGCAGGCCTGCCCCCACTAACAGGCTTCCTACCAAAGTGACTCATTATCCA
AGAGCTCTCTAAGCAGGAAATAGCCGCCTCCGCCACAATCATCGCCGTCCTATCCCTGCTAGGCTTATTCTTC
TACCTCCGCCTAGCTTACTACTCAACAATCACCCTACCACCTAGCCCCACAAACCACATAAAACAATGGTACA
CCAATAAACCCACAAACACCATAATCGCTGCACTTTCCTCCATATCAACCCTCCTACTTCCACTGTCCCCCAT
AATCCTTGCCTCCATCTAA
Anthracothorax prevostii   EU983409.1
ATGAACCCCTATGCCAAGCTAACATTCCTTTTAAGCCTCACACTAGGGACAACTATCACCATCTCCAGCAATC
ACTGAATAATAGCCTGGTGCGGACTTGAAATCAACACCCTAGCCATCATTCCTCTAATCTCAAAATCCCACCA
CCCCCGAGCCATCGAAGCAGCAATTAAATACTTTTTAGTTCAAGCCGCCGCCTCCACACTACTACTATTCTCA
AGTATGGTCAATGCCTGATACACGGGCCAATGAGACATCACCCAATTAAATAACTCTACATCATCCATGCTTC
TAACAGCAGCTGTAGCAATAAAACTTGGCCTAGTCCCATTTCACTTTTGATTCCCAGAGGTACTACAAGGCTC
CCCCCTAGTCACTGCCATACTCCTATCAACAGTAATGAAATTCCCCCCAATCTCCATCCTATTCCTCACATCA
CACTCGCTAAACCCCGCCCTACTGACTACAATAGCAATTGCCTCAGCCACACTAGGAGGGTGAATAGGCCTAA
ACCAAACACAAACCCGAAAAATCCTAGCCTTCTCATCAATCTCCCATCTAGGATGAATAACCATCACTCTCAT
TTATAACCCCAAACTGACACTGTTAACTTTCTACTTATACTGCATTATAACTAGTGCTGTCTTCCTAACCCTT
GACACTACAAAATCTCTTAAACTATCAACAATAATAACCTCCTGGGCAAAAATACCTACACTGAACGCAGCCT
TAATAATGACACTACTGTCATTAGCAGGCTTACCCCCCCTAACAGGATTCCTGCCCAAGTGACTCATCATCCA
AGAGCTATCCAAGCAAGAAGTAACTGCCTCTGCCACAATTATCGCCATTTTATCTCTATTGGGCCTATTTTTC
TACCTCCGTTTAGCCTACTACGCAACAATTACACTCCCACCCAACCCAACAAACCACATAAAGCAATGGCACA
CCAACAAGCCGTCAAACACCTCAATCGCCATGCTAACCTCCTTATCAGCTCTCTTACTCCCCCTATCCCCAAT
AATCCTCGCCTCTATTTAG
Colibri thalassinus   EU042544.1
ATGAATCCACACGCCAAACTAGCATTCTCTCTCAGCCTCATGCTAGGAACAACCATCACCATTTCAAGCAACC
ACTGGGTAATAGCCTGGGCTGGACTTGAAATTAATACCTTAGCCATCATCCCGCTTATCTCAAAATCCCACCA
CCCCCGAGCCATTGAGGCAGCAATCAAATACTTCCTAGTCCAAGCCGCTGCCTCCACACTACTACTCTTCTCA
AGCATAATCAATGCTTGGCACTTAGGACAGTGAGACATTACTCAACTTAATCACCCCACGTCGTCCTTATTAC
TAACAACAGCAATCGCAATAAAACTAGGCCTAGTCCCATTCCACTTCTGATTCCCAGAAGTCCTCCAAGGCTC
TCCGCTTACCACTGCAATACTGCTATCAACAGTAATAAAACTCCCCCCCATCACTATCCTCTTTCTCACATCC
CACTCACTAAACCCCGCCCTACTCACTACAATAGCAATCGCCTCCGCCGCACTGGGGGGATGAATGGGACTCA
ACCAAACACAAATTCGAAAAGTCCTGGCCTTCTCCTCAATCTCGCACCTGGGATGAATGGCCATTACCCTAGT
CTACAGCCCAAAACTAGCACTAATGGCCTTTTACCTCTACTGTATCATAACTTGCGCAATTTTCCTAACCCTC
CACACAACAAAATCTCTAAAACTATCTACAATAATAACCTCCTGAACAAAAATGCCTACACTTAATGCAGCCC
TGATAATGACACTACTATCACTAGCAGGCCTACCCCCGCTAACTGGATTCTTGCCCAAATGGCTCATTATCCA

AGAACTCTCTAAGCAAGAAATAACAATCTCCGCCACAATTATCGCCATCCTATCTCTACTAGGCCTATTCTTT
TACCTGCGCCTAGCTTACCACTCAACAATCACCCTCCCACCAAGCCCCACAAACTACATGAAACAGTGACACA
TCAACAAACCTACGAGCACTTCAATCGCTGTGCTCACCTCTCTATCAGCCCTCCTTCTTCCTCTGTCCCCAAT
AATCCTCGCCTCCATTTAG

Eugenes fulgenes AY830481.1

ATGAACCCCTACGCTAAACTAACATTCTCCATAAGCCTCATGTTAGGAACAACCATCACCATTTCAAGTAGCC
ATTGGGTATCCGCCTGAGCCGGACTTGAAATCAACACTCTAGCCATCATTCCGCTTATTTCACAATCCCACCA
CCCCCGAGCCATTGAGGCAGCAATCAAATACTTCCTTGTCCAAGCTGCTGCCTCCACCTTACTACTATTCTCA
AGTATAGTCAATGCCTGACACACAGGACAATGGGACATCACTCAACTAAGCTACCCCACATCATCCCTACTAC
TAACCACGGCAGTAGCAATAAAACTTGGCTTAGTACCATTCCATTTCTGATTTCCAGAAGTCTTACAAGGATC
CCCCCTAAGCACAGCCATACTATTATCCACAGTAATAAAATTCCCCCCAATCACCATTTTATTCCTCACATCT
CACTCCTTAAACCCAACCCTCCTCACCATAATGGCACTCACTTCAGCTGCCCTAGGGGGATGAATAGGACTAA
ACCAGACACAAATTCGAAAAGTCCTAGCTTTCTCATCAATCTCCCACCTCGGATGAATAACCATTGTCCTTAT
CTATAACCCAAAATTAACATTAATAACCTTCTACCTATACTGCATTATAACCAGCGCCATCTTTTTAACCCTT
AACACTACAAAATCCCTCAAACTGTCAACAATAATAACCTCCTGAACAAAAGCTCCAATACTCAATACTACAT
TAATAATAACACTTCTCTCACTAGCAGGTTTACCCCCATTAACCGGATTTTTACCCAAATGACTCATCATCCA
AGAACTCTCCAAACAAGAAATATCCACCTCTGCTACAGTTATTGCCATCCTATCCTTATTGGGCCTATTCTTC
TACCTCCGCCTAGCTTATTACTCAACAATCACCCTACCACCAAACCCCACAAACCACATAAAACAATGGTACA
CTAATAAACCTGCAAGCAGCCTAATCGCCATGCTCTCCTCCCTGTCAGCCCTCCTACTCCCACTATCCCCAAT
AATCCTCGCCTCCATTTAA

* Due the available sequences on GenBank for Wedge-tailed Sabrewing (Pampa curvipennis) are partial (KC858426 393 bp and KC858427 393 bp), we used a complete sequence of gene ND2 of closely related species Long-tailed Sabrewing (Pampa excellens).
